# Two alternatively-spliced human nebulin isoforms with either exon 143 or exon 144 and their developmental regulation

**DOI:** 10.1038/s41598-018-33281-6

**Published:** 2018-10-24

**Authors:** Le Thanh Lam, Ian Holt, Jenni Laitila, Mubashir Hanif, Katarina Pelin, Carina Wallgren-Pettersson, Caroline A. Sewry, Glenn E. Morris

**Affiliations:** 1 Wolfson Centre for Inherited Neuromuscular Disease, RJAH Orthopaedic Hospital, Oswestry, SY10 7AG UK; 2 Institute for Science and Technology in Medicine, Keele University, Keele, UK; 3 The Folkhälsan Institute of Genetics, Department of Medical and Clinical Genetics, University of Helsinki, Helsinki, Finland; 4 Faculty of Biological and Environmental Sciences, Molecular and Integrative Biosciences Research Programme, University of Helsinki, Helsinki, Finland; 5 Dubowitz Neuromuscular Centre, Institute for Child Health and Great Ormond Street Hospital, London, UK

## Abstract

Nebulin is a very large protein required for assembly of the contractile machinery in muscle. Mutations in the nebulin gene *NEB* are a common cause of nemaline myopathy. Nebulin mRNA is alternatively-spliced so that each mRNA contains either exon 143 or exon 144. We have produced monoclonal antibodies specific for the regions of nebulin encoded by these two exons, enabling analysis of expression of isoforms at the protein level for the first time. All antibodies recognized a protein of the expected size (600–900 kD) and stained cross-striations of sarcomeres in muscle sections. Expression of exon 143 is developmentally-regulated since newly-formed myotubes in cell culture expressed nebulin with exon 144 only; this was confirmed at the mRNA level by qPCR. In fetal muscle, nebulin with exon 143 was expressed in some myotubes by 12-weeks of gestation and strongly-expressed in most myotubes by 17-weeks. In mature human muscle, the exon 144 antibody stained all fibres, but the exon 143 antibody staining varied from very strong in some fibres to almost-undetectable in other fibres. The results show that nebulin containing exon 144 is the default isoform early in myogenesis, while regulated expression of nebulin containing exon 143 occurs at later stages of muscle development.

## Introduction

Nebulin is a large protein expressed mainly in the skeletal muscle sarcomere^1–3^. It has a modular structure, with more than 200 simple repeats, each binding to actin along the length of the thin filament^1,2^. The most important roles of nebulin are to act as a thin filament stabiliser and in defining Z-disc width^4–7^. Although all known exons in the *NEB* gene could theoretically encode a protein of 8,525 amino-acids^8^, numerous alternative splicing events typically result in smaller proteins in the range 600–900 kD^9,10^. Whereas most of these alternative splicing events simply remove exons from the nebulin mRNAs, all nebulin mRNAs contain either exon 143 or exon 144 but never both^2^. In the central region of nebulin, the 200+ simple repeats are further organised into 26 different super-repeats. The mutually exclusive exons 143 and 144 are found in S21 and define S21a and S21b respectively^1,2^.

The amino-acid sequence of nebulin is highly conserved among species. The sequence encoded by exon 143 shows complete homology between mouse, rat, and human. However, S21a and S21b differ in charge and hydrophobicity^2^, suggesting different functional roles for the alternative isoforms. The S21 super-repeat is just outside the Z-disc anchorage region. It contains the binding site for KLHL40, a muscle-specific Kelch protein that also binds leiomodin-3 (LMOD3) and stabilizes thin filament assembly^3,11^. Mutations in several proteins, including nebulin, KLHL40^11^ and LMOD3^12^, can cause nemaline myopathy, a congenital disorder of muscle characterized by the presence of nemaline bodies, which are aggregates of thin filament proteins^13,14^. Mutations in two other Kelch genes, those for KLHL41 and KBTBD13, also cause forms of nemaline myopathy^15^.

We have produced eleven monoclonal antibodies for the S21 region, including three that are specific for nebulin protein encoded by exon 143 or exon 144. For the first time, these new antibodies have enabled a study of the two nebulin isoforms during human muscle development at the protein level. Immunolocalization has enabled us to follow nebulin isoform expression in different cell and fibre types at several stages of human muscle development, from early myogenesis in cell culture, through fetal stages, to mature skeletal muscle. We suggest that S21a (nebulin with exon 143) has functions that are required at later stages of muscle development.

## Results

### New monoclonal antibodies confirm the existence of nebulin isoforms at the protein level.

BALB/c mice were immunised with recombinant fragments of nebulin encoded by exons 138–143 or 138-(143)144. The fragments were produced from pET plasmids as fusion proteins with thioredoxin. The use of fragments larger than exons 143 or 144 alone was employed to increase their immunogenicity, since the amino-acid sequence of exon 143 is identical in man and mouse.

Our initial hybridoma screening was based on ELISA with the two fragments, with the aim of selecting mAbs that recognised 138–143, but not 138–144, and vice-versa. The first attempt produced only mAbs that were directed against exons 138–142 common to both fragments (Table 1A).

**Table 1:** New monoclonal antibodies against human nebulin.

Antibody	Epitope (encoding exons)	Sub-type
**(A)**		
NEB-6B5	ex140	IgG1
NEB-9C4	ex140	IgG1
NEB-8D2	ex140	IgG1
NEB-9A11	ex138-142	IgG1
NEB-10A7	ex138-142	IgG1
NEB-9D9	ex138-142	IgG1
NEB-8G3	ex138-142	IgG1
NEB-7D5	ex138-142	IgG1
**(B)**		
NEB-3F4	ex143	IgG1
NEB-6E6	ex143	IgG1
NEB-9H1	ex144	IgG1

**Figure 1:**
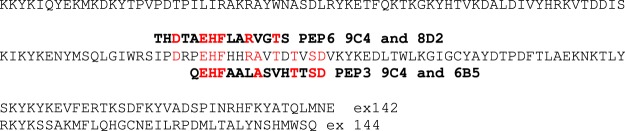
Phage display epitope mapping of three nebulin mAbs. Biopanning with a mixture of the 11 mAbs succeeded in selecting from the library, 2 phage-displayed peptides recognized by 3 of the mAbs. The peptide sequences obtained by sequencing the phage DNA inserts are aligned with the protein sequence encoded by nebulin exons 138-139-140-141-142-144 and they show an excellent match of 6 or 7 amino-acids within each of the two overlapping 15-mer peptides. Peptide PEP3 was recognized by both NEB-6B5 and NEB-9C4, whereas PEP6 was recognized by both NEB-9C4 and NEB-8D2. Both peptides are encoded by exon 140 of the *NEB* gene.

**Figure 2:**
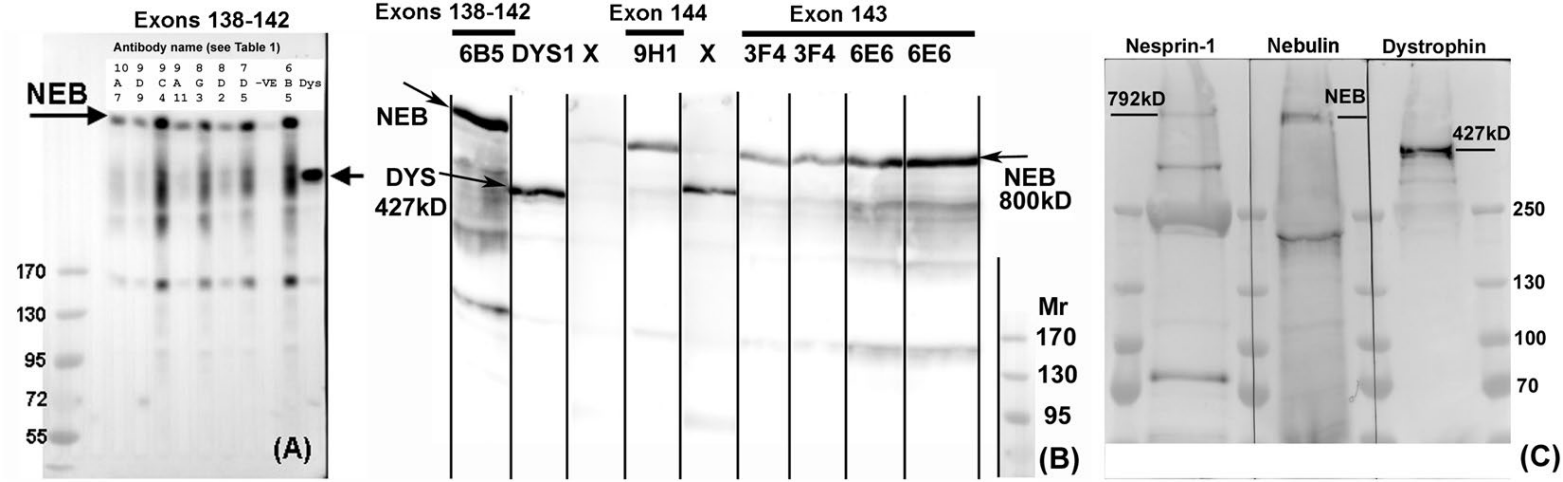
Monoclonal antibodies against different nebulin epitopes recognize a protein of ca 800 kD on western blots. (**A**) Different mAb supernatants were applied to a strip blot of human muscle extract using a miniblotter. Lanes 1–7 and 9 are the eight mAbs in Table 1A that recognize exons 138–142. Lane 8 is a negative control and lane 10 is MANDYS1 mAb which recognizes 427 kD dystrophin. (**B**) In this case, the blot was cut into strips which were exposed to different mAb supernatants separately. The first lane is NEB-6B5 from lane 9 in (**A**) and lane 2 is MANDYS1 anti-dystrophin marker. NEB-9H1 is the exon 144 mAb and the two flanking lanes labelled “X” are two supernatants from the hybridoma fusion that were not specific for nebulin and not cloned; these are followed by two sub-clones each of the exon 143 mAbs, NEB-3F4 and NEB-6E6. (**C**) A freshly-made extract of human muscle was loaded into 3 lanes separated by pre-stained Mr markers. The blot was cut vertically into 3 sections through the Mr lanes and each section was developed with MANNES2A anti-nesprin-2 (792 kD), NEB-6B5 or MANDYS1 anti-dystrophin (427 kD), as described in Methods. The largest prestained Mr marker (250 kD) is also shown. The prestained marker images and the chemiluminescent images were exactly superimposed using the Photoshop “Apply Image” tool. In (**B**,**C**), the lanes were cut from the same blot, reassembled in sequence after development and exposed together to obtain chemiluminescent images; (**A**) is a single miniblotter blot.

**Figure 3:**
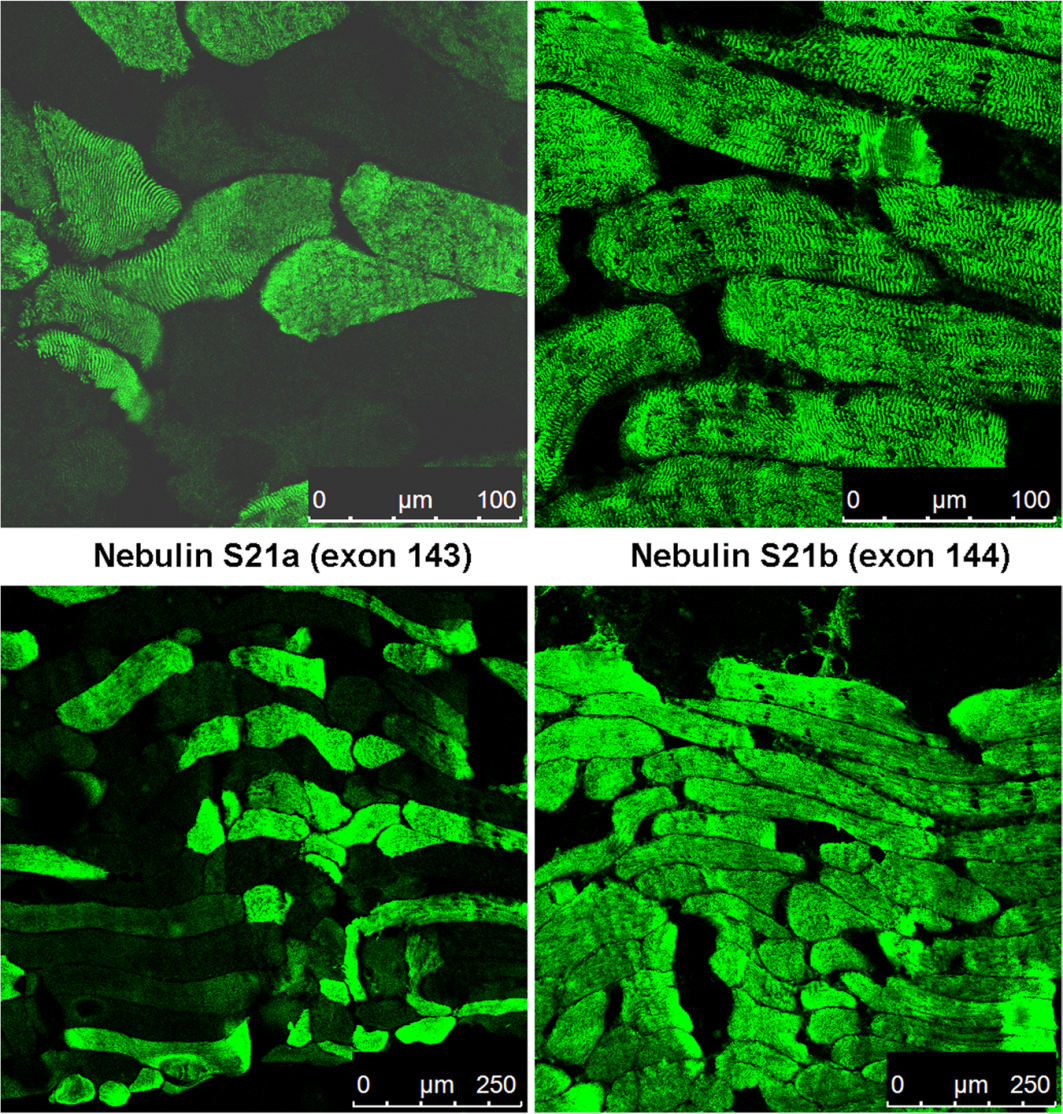
Nebulin with S21b (exon 144) is found in cross-striations of all muscle fibres, but nebulin with S21a (exon 143) is found in some fibres only. Muscle sections (quadriceps) were labelled with either an exon 143 mAb (NEB-3F4) or the exon 144 mAb (NEB-9H1) and confocal images were acquired with either high magnification (63x objective: upper two frames) or at lower magnification (20x objective: lower two frames). For NEB-3F4, fibres are variably labelled strongly, weakly or not at all.

It was possible to map the epitopes recognised by 3 of these mAbs using phage-displayed peptide libraries and they were binding to regions encoded by exon 140 (Fig. 1). Other mAbs did not select peptides from the library and could not be mapped in this way. This panel of 6 mAbs all recognised a band on western blots of human muscle consistent with the expected size of nebulin (>427 kD dystrophin) (Fig. 2A) and stained sarcomeric bands in longitudinal skeletal muscle sections (Fig. 3A). To produce the desired exon 143/144 mAbs, we screened with new fusion proteins expressing exon 143 only or exon 144 only and produced two mAbs specific for exon 143 (NEB-3F4 and NEB-6E6) and one mAb specific for exon 144 (NEB-9H1) (Table 1B). The two exon 143 mAbs stained sarcomeric cross-striations in only a subset of fibres and with variable intensity, whereas the exon 144 mAb stained the striations in all fibres with little variation in intensity (Fig. 3B). All three mAbs stained a similar nebulin band on western blots of normal adult human muscle (Fig. 2B). Lower Mr bands on the blot are likely degradation products of nebulin. Figure 2C shows that the largest nebulin band is similar in size to nesprin-2 (792 kD) or slightly smaller. We have labelled it “800 kD” as an approximation, since it is clearly smaller than the predicted “unspliced” size (980 kD) of the largest, full-length nebulin sequence^8^, as expected^9,10^.

### Early myotubes in cell culture have only nebulin with exon 144, but myotubes in fetal muscle at 12 weeks of gestation also express nebulin with exon 143

**Figure 4:**
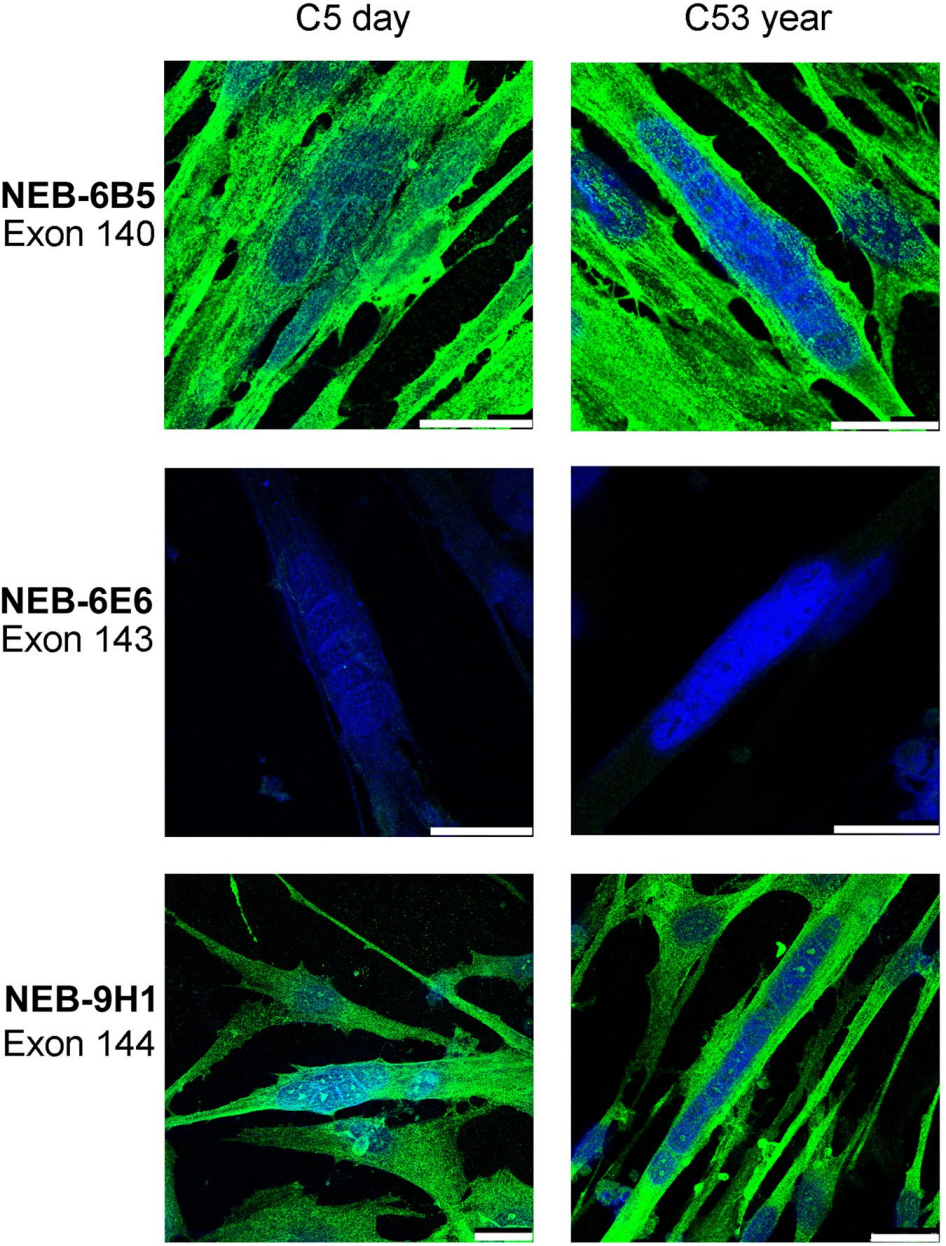
Multinucleate human muscle myotubes in cell culture only produce the S21b (exon 144) isoform of nebulin and not the alternatively-spliced S21a isoform (exon 143). Two different immortalised cell lines were used, one derived from a 5-day old infant (left column) and one from a 53-year-old donor (right column). The exon 143 mAb shown is NEB-6E6 but identical results were obtained with NEB-3F4. The size bars are 25 microns in each frame.

Cultures of human myotubes from immortalised clonal cell lines were examined 4 days after induction of myoblast fusion by removal of serum from the culture medium. At this stage, over 60% of nuclei are within multinucleate myotubes, levels of muscle-specific creatine kinase (M-CK) are high and myosins (fast, slow and neonatal) are already present (data not shown). Nebulin was barely detectable with exon 143 mAbs, but the exon 144 and exon 140 mAb showed a uniform staining of the myotube cytoplasm (Fig. 4). The same result was obtained with two different cell lines, from donors aged 5 days or 53 years.

**Figure 5:**
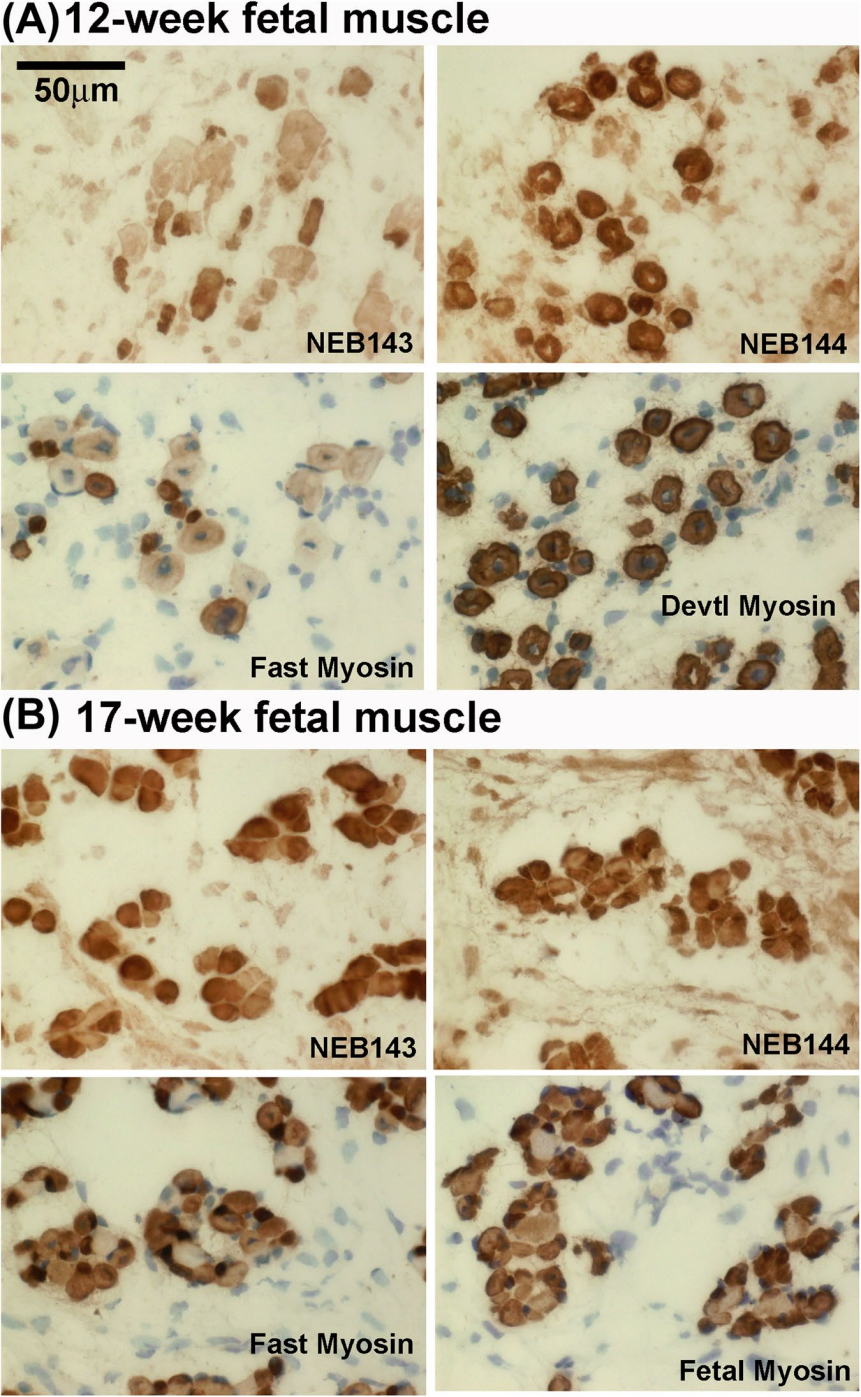
Expression of nebulin protein S21a (with exon 143) increases progressively during human fetal muscle development, becoming as strong as S21b (exon 144) in some fibres by 17-weeks of gestation. Frozen sections of (**A**) 12-week or (**B**) 17-week gestation fetal muscle were stained with mAbs against S21a (NEB-3F4 or “NEB143”), S21b (NEB-9H1 or “NEB144”) or fast, fetal and developmental myosin isoforms as indicated. Myosin stainings were counter-stained for nuclei with haematoxylin. Note the central nuclei in fetal myotubes. Surrounding connective tissue, revealed by haematoxylin-stained nuclei, is not stained by any of the mAbs. The 50 micron size bar in the first frame applies to all 8 frames.

qPCR confirmed that only 1% of nebulin mRNAs in myotube cultures had exon 143, whereas in adult muscle up to two-thirds of the mRNA had exon 143 (Table 2). Levels of nebulin mRNA containing exon 143 are much higher in fetal muscle, and higher again in adult muscle, compared with cultured myotubes (Table 2). All myotubes in sections of human fetal muscle at 12 weeks gestation expressed developmental myosin strongly while fast myosin was expressed less strongly and more variably (Fig. 5A). Similarly, S21b nebulin (exon 144) was found in all fibres but with only a little variability. Staining fetal muscle of 12 weeks gestation for S21a nebulin (exon 143) gave a pattern similar to fast myosin staining; thus, many of the larger myotubes are less strongly-stained for both S21a and fast myosin than smaller myotubes (Fig. 5A). The larger ones may be primary myotubes and the smaller ones may be the secondary myotubes that appear a little later in human myogenesis.

**Table 2:** Relative expression by qPCR of mRNAs for nebulin isoforms in human cultured cells and skeletal muscle. Three different immortalised myoblast lines were used and are identified by the age of the original muscle donor. Derivation of “relative expression” is described in Methods. The percentage exon 143 values in parenthesis are (ex143/[ex143 + ex144]) × 100. Values are mean ± SD of 3 determinations.

Tissue Source	Relative expression	
	Ex143	Ex144
Myoblasts (5 days)	<1	<1
Myotubes (5 days)	4.7 ± 0.4 (0.4%)	1160.7 ± 154.0
Myoblasts (25 years)	<1	3.2 ± 0.9
Myotubes (25 years)	49.9 ± 3.7 (1.05%)	4721.1 ± 748.4
Myoblasts (53 years)	<1	16.8 ± 2.8
Myotubes (53 years)	48.4 ± 9.3 (1.1%)	4310.3 ± 544.3
Fetal skeletal muscle (MRC Biobank)	904.8 ± 194.4 (16%)	4688.3 ± 652.8
Adult skeletal muscle (RJAH, 18 years)	32177.1 ± 2192.3 (72%)	12372.9 ± 3298.5

By 17 weeks of fetal development, staining for S21a (exon 143) of the larger myotubes was stronger and the differences between 143 and 144 were less evident (Fig. 5B). Some very small myotubes expressing fast and fetal myosin are evident with mAb NEB-9H1 (exon 144) but not with mAb NEB-3F4 (exon 143) (Fig. 5B). These myotubes may be a subset of secondary myotubes sometimes referred to as tertiary myotubes^16^. From the results with these two fetal muscle samples, it seems that nebulin with exon 143 has appeared by 12 weeks gestation and may have increased further by 17 weeks.

The results suggest that the first nebulin produced during myogenesis contains only S21b (exon 144) super-repeat and that alternative splicing to produce additional nebulin molecules with the S21a (exon 143) super-repeat occurs at later stages of muscle differentiation and development.

### In mature human quadriceps muscle, fibres expressing fast myosin usually express S21a nebulin (exon 143)

**Figure 6:**
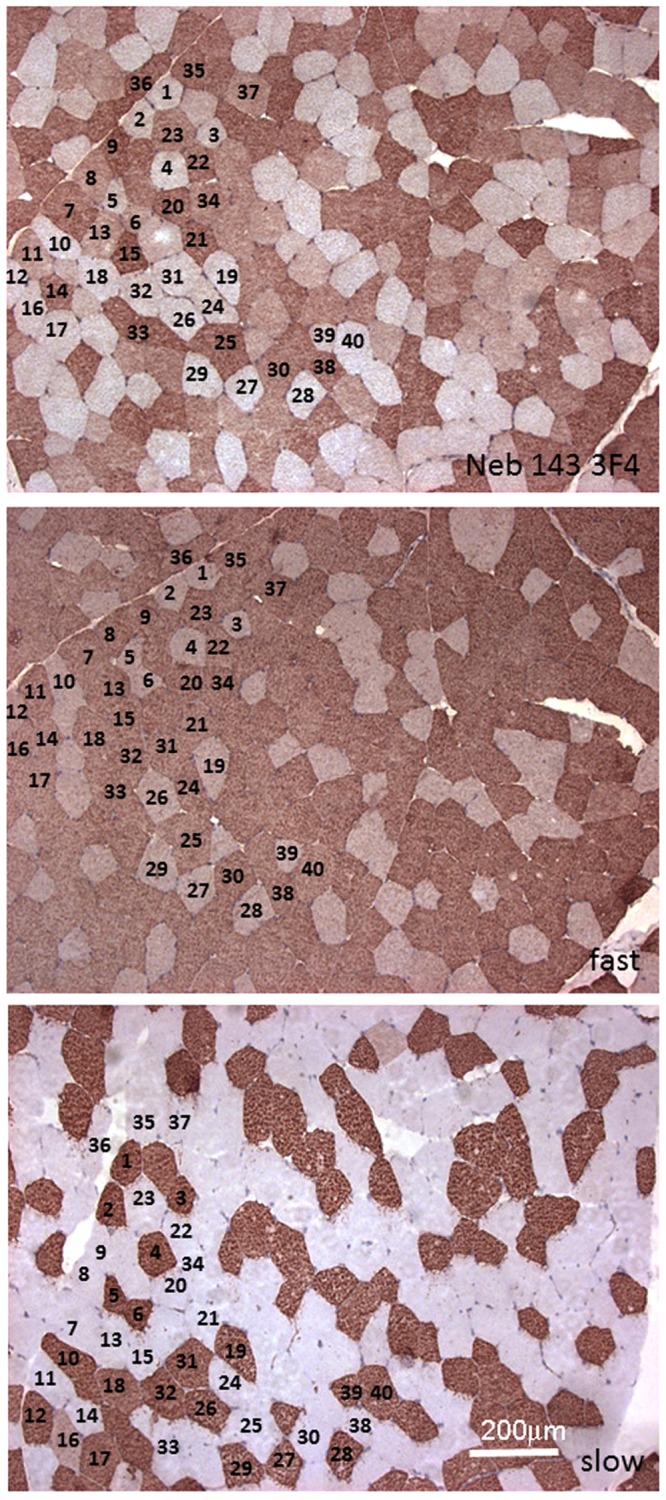
Fibres in human quadriceps muscle expressing the fast myosin isoform are usually also stained by the mAbs specific for the S21a isoform (NEB-3F4). Serial sections from a quadriceps sample were immunostained by the peroxidase method with mAbs against nebulin encoded by exon 143, fast myosin or slow myosin (see Methods). Some of the fibres are numbered to enable easier comparison of the three serial sections. Most fibres are either “fast” or “slow” but some “hybrid” fibres also occur (e.g. 17 and 40). Most fibres with fast myosin are also labelled with NEB-3F4. The 200 micron size bar in the bottom frame applies to all 3 frames.

We tried to determine whether the variable expression of exon 143 between adjacent muscle fibres seen in Fig. 3 is related to any other property that shows this “fibre-typing” effect, the best known being the mixture, in various proportions, of fast-twitch and slow-twitch fibres of which most muscles are comprised. Serial sections of a human muscle were stained with mAbs against nebulin (143 or 144) or myosin (fast or slow). We did not attempt to distinguish between IIA and IIX fast myosin^17^. In Fig. 6, some of the fibres (40 out of 100 counted) are numbered to identify the same fibre in each of the serial sections. Two additional quadriceps biopsies from two different subjects were analysed in the same way and Table 3 shows the results from these 3 experiments (the biopsy for Fig. 6 is “Quads 3” in Table 3). The great majority (93.3%) of fibres expressing fast myosin were stained by NEB-3F4 mAb (exon 143), but 24.1% of the fibres staining for slow myosin were also stained by NEB-3F4 mAb. Thus, fast fibres usually express S21a nebulin (exon 143) while slow fibres may express either S21a or S21b.

**Table 3:** The association between expression of fast myosin and nebulin exon 143 is significantly non-random. Logistical Regression Analysis (Logit Method) for a binary dependent variable was used to determine 95% confidence intervals (CI). The method gives the probability that association between fast myosin and nebulin exon 143 is random (50%).

Biopsy Sample	No. of fibres counted	% fast fibres	% fast fibres exon 143+	% slow fibres exon 143+
Quads 1	62	63	92	29
Quads 2	110	55	98.4	41
Quads 3	100	53	84	10.6
Mean			93.3%	24.1%
95% CI			67.7–100%	6.3–64.5%

Even with this very small sample number (N = 3), the association between fast myosin and exon 143 expression was significant [p < 0.05] (Mean % of fast fibres that are exon 143+ = 93.3%; 95% CI range 66.7–100%), but no significant association between slow fibres and absence of exon 143 expression could be demonstrated at this probability level (Mean % of slow fibres that are exon 143+ = 24.1%; 95% CI range 6.3–64.5% [p > 0.05]).

## Discussion

This study has proven, for the first time at the protein level, the existence of two alternatively-spliced nebulins, isoforms S21a with exon 143 and S21b with exon 144, previously known from mRNA studies only. Although each single nebulin mRNA contains either exon 143 or exon 144, and never both^2^, we have shown that all human multinucleate muscle fibres examined contain nebulin isoform S21b (exon 144) while some of them co-express nebulin S21a (exon 143). Because of the multinuclearity of the myofibres, it is not possible to say whether both alternative splicings occur within a single myofibre nucleus, or whether some myofibre nuclei express only exon 143 and others only exon 144. The nebulin protein sequence encoded by exon 143 is highly-conserved across species, suggesting that it may have a special function, such as specific interaction with another protein. For example, the binding site for KLHL40 is within S21, but its precise location is not known^11^. The existence in adult muscle of fibres containing only S21b (with exon 144) shows that the inferred special function of S21a is not required in all fibres. The expression of S21a in addition to S21b in some fibres may explain may explain why “fibre-typing” effects were observed in previous studies when sections were labelled with polyclonal pan-nebulin antibodies^18^.

Cultured myotubes *in vitro* contained only nebulin with exon 144 (Fig. 4), but by the second trimester of fetal development *in vivo*, most myotubes contained both nebulins (Fig. 5), suggesting developmental regulation of some kind. At 12 weeks gestation, the smaller fetal myotubes tended to be stained more strongly for both exon 143 and fast myosin than the larger, primary, fetal myotubes (Fig. 5A). However, at 17 weeks gestation, some very small myotubes with fast myosin (possible tertiary myotubes^16^) were more strongly stained with the exon 144 mAb (Fig. 5B). These observations, together with variation between individual adult fibres (Fig. 6), shows that expression of S21a (exon 143) is not the result of a simple or irreversible developmental switch. Splicing of primary RNA transcripts into mRNAs is determined by cis-acting sequences in adjacent introns and by trans-acting factors (proteins or micro-RNAs) which may themselves be regulated by epigenetic changes during development. It remains to be determined whether the trans-acting factors affecting S21a expression are independently-regulated or linked to a specific gene-switching event during muscle development.

This first study at the protein level differs from earlier mRNA studies, since we are sampling only a small area of a human muscle section whereas mRNA was extracted from whole muscles. The latter gives a broader picture, but the former enables a direct appraisal of the role of fibre-types. Whereas mRNA studies showed that alternative splicing is not determined by the average fast/slow composition of muscle^19^, our ability to study splicing in individual fibres using antibodies has shown that it does depend to some extent on fibre-type, though it may not be directly-related to myosin isoform expression. The present human studies confirm and extend the work of Donner *et al*.^19^ on alternative splicing of mouse nebulin exons 127 and 128 (murine equivalent of human exons 143 and 144) in different mouse muscles at different ages up to 6 weeks (near-adult in the mouse) by extracting RNA from whole mouse muscles. This mouse study found considerable differences between different muscles; thus, many muscles, including quadriceps and gastrocnemius, had mainly exon 128 by 6-weeks, though they expressed a lot of exon 127 at earlier stages, while some muscles (longus capitis) expressed almost exclusively exon 127 and others (diaphragm, masseter) expressed almost exclusively exon 128 at all ages. In human quadriceps, most fast fibres expressed exon 143 and most slow fibres did not. It will be necessary to study other human muscles in order to determine whether alternative splicing varies with human muscle type, as it does between mouse muscles. Some other alternative splicings of human nebulin mRNA have been shown to occur only in soleus muscle, and not in other muscle types^9^, suggesting that post-transcriptional effects on nebulin expression may often be muscle-type-specific.

Future studies of biopsies from patients with neuromuscular disorders, including nemaline myopathy, and of a variety of muscles in addition to quadriceps, will be necessary to clarify the effects of muscle-type and disease state.

## Materials and Methods

### Ethical Approvals

The project was approved by the RJAH Hospital Research Committee. Animal procedures for mouse monoclonal antibody production were carried out under UK Home Office Licence and approved by Keele University Animal Welfare and Ethical Review Body. All human tissue studies complied with the UK Human Tissue Act (2006). Use of donor muscle tissue from routine orthopaedic surgery with informed consent was approved by the UK NHS Review Body (NRES North West No 11/NW/0875). Other human muscle samples were obtained from the MRC Centre for Neuromuscular Disease Biobank, London. Parental or legal guardian informed consent was obtained for participants under the age of 16 years.

### Production of recombinant immunogens

The DNA fragments containing nebulin exons 138–143 or 138–144 were obtained from thigh muscle RNA by reverse transcription PCR, and cloned into pET32a-vector^20^. Plasmid constructs containing the correct insert were identified by Sanger sequencing. For screening, the cDNA fragments containing exon 143 or exon 144 alone, obtained from the pET32a construct by PCR, were cloned into pGEX4T-1 vector for expression as GST fusion proteins. All constructs were transformed into *E. coli* BL21 (DE3) and the expression was induced with IPTG (0.5 mM final concentration).

### Production of monoclonal antibodies

Monoclonal antibody production was performed as previously described^21^. Spleen cells from hyper-immune BALB/c mice were fused with the Sp2/O mouse myeloma cell line using PEG1500 and plated in 960 wells of microtitre plates. After 10 days, the hybridomas were screened by ELISA, western blot and immunocytochemistry. Positive wells were cloned twice by limiting dilution.

### Phage-displayed Random Peptide Library

Epitope mapping with a phage-displayed random 15-mer library was carried out according to a detailed protocol^22^. Briefly, antibody coated onto plastic dishes was used to select phage particles from a 15-mer peptide library in phage f88–4, maintained in the K91Kan strain of *E*. *coli* and generously supplied by G.P. Smith (University of Missouri). After 2–3 rounds of selection, individual phage-infected bacterial colonies positive for the antibody target on western blots were isolated and the expressed peptide sequence was identified by sequencing the phage DNA with a suitable primer (5′-AGTAGCAGAAGCCTGAAGA-3′).

### Muscle cell culture

Clonal myoblast cell lines, immortalized by transduction with human telomerase reverse transcriptase (hTERT) and cyclin-dependent kinase-4 (Cdk4)^23^, were provided by Dr V. Mouly, Institut de Myologie, Paris. They were cultured in skeletal cell growth medium (Cat No: C-23060; PromoCell GmbH, Heidelberg, Germany) containing supplement mix (Cat No: C-39365; PromoCell) with 20% Fetal Bovine Serum (Cat No: 10270; Gibco, ThermoFisher, Paisley, UK). Differentiation was induced at 80% confluency by washing the adherent myoblasts in medium lacking serum and then culturing in DMEM (Cat No: 31966-021; Gibco, ThermoFisher) supplemented with Insulin (1721 nM), Transferrin (68.7 nM), Selenium (38.7 nM) (ITS-X; Cat No: 51500-056; Gibco, ThermoFisher) and Penicillin-Streptomycin. After a further 4 days of cell culture, over 60% of the cells had fused into myotubes.

### Immunolabelling of muscle sections using peroxidise

Immunolabelling was performed on unfixed 5–6 micron thick sections of muscle frozen in isopentane cooled in liquid nitrogen. Sections were immunolabelled with nebulin primary antibodies (diluted 1:3 in PBS buffer) for 1 hour and visualised using the X-cell Plus Polymer kit (A.Menarini Diagnostics, Winnersh-Wokingham, UK) followed by 3, 3′ diaminobenzidine and peroxidase. Counterstaining was with haematoxylin. After labelling and counterstaining, sections were dehydrated, cleared in xylene and mounted in DPX. Monoclonal antibodies (mAbs) against myosin heavy-chains from Leica Biosystems were: Fast myosin (NCL-MHCf), Slow myosin (NCL-MHCs), Neonatal myosin (NCL-MHCn) and Developmental myosin (NCL-MHCd). They were used according to supplier’s recommendations and labelled with the Menarini kit described above. The muscle biopsies (quadriceps) were from a 15-year old male cerebral palsy patient, the MRC Biobank or a mild myopathy patient (13 year old female).

### Immunofluorescence microscopy

Immunolabelling was performed on cells cultured on coverslips, fixed with 50:50 acetone-methanol and washed with PBS, or on muscle frozen sections. Monoclonal antibodies in culture supernatants were diluted 1:3 in PBS and incubated on the specimen for 1 hour. Following washing, incubation was continued with 5 µg/ml goat anti-mouse ALEXA 488 (Molecular Probes, Eugene, Oregon, USA) secondary antibody diluted in PBS containing 1% horse serum, 1% fetal bovine serum and 0.1% BSA, for 1 hour. DAPI (diamidinophenylindole at 200 ng/ml) was added for the final 10 minutes of incubation to counterstain nuclei before mounting in Hydromount (Merck). Sequential confocal scans performed with a Leica TCS SP5 spectral confocal microscope (Leica Microsystems, Milton Keynes, UK).

### SDS-polyacrylamide gel electrophoresis and Western blotting

Human muscle tissue was extracted in: 50 mM Tris pH 6.8, 1% EDTA, 10% SDS, 5% beta-mercaptoethanol, 10% glycerol with protease inhibitors. After the addition of bromophenol blue and after boiling, the extract was loaded, either as a single strip or in formed wells, and subjected to SDS-PAGE using 4 to 12% polyacrylamide gradient gels and transferred to nitrocellulose membranes (Protan BA85, Whatman). After blocking non-specific sites with 5% skimmed milk protein, membranes were incubated for 1 hour with monoclonal antibody (1/50), followed by washing and incubation for 1 hour with peroxidase-labelled rabbit anti-mouse immunoglobulins (1/1000, Dakopatts, Denmark). Antibody reacting bands were visualized with West Pico or West Femto chemiluminescent detection systems (Pierce, Thermofisher). Prestained Mr markers were EZ-Run 10–170 kD or PageRuler Plus 10–250 kD (both Thermofisher).

### qPCR

Total RNA was prepared from cultured cells and from skeletal muscle samples using RNeasy Plus Mini Kit (Qiagen) and quantified with a NanoDrop ND-1000 spectrophotometer. Fetal muscle was obtained from the MRC Centre for Neuromuscular Disease Biobank, London. Adult muscle (18 year male) was obtained during routine surgery at RJAH Orthopaedic Hospital, Oswestry (with informed consent and ethical approval). Total RNA (maximum of 2.5 µg in a 20 µL reaction) was reverse transcribed (SuperScript VILO cDNA Synthesis Kit; Applied Biosystems) and then diluted with sterile water, in order to achieve the cDNA equivalent of 10 ng total RNA for each 20 µL reaction in the qPCR plate. Primers were: Ex143F: 5′GCTGATTATGAGCAGCGGAAAG or Ex144F: 5′GGATGTAATGAAATTCTGCGT in combination with Ex145R: 5′TCGCTGATTTGTTTGTTGACTT.

Relative quantitative PCR was performed using SYBR green detection in an ABI 7500 Real Time PCR system (Applied Biosystems). Reaction wells contained 10 µL SYBR Select Master Mix (Applied Biosystems), 1.5 µL cDNA, 300 nM Forward and 300 nM Reverse primers in a final volume of 20 µL. For each preparation of cDNA, each target sequence was amplified along with two endogenous controls (Beta-actin and GAPDH). Quantitation of target transcripts relative to the two endogenous reference transcripts were calculated by the 2^−∆CT^ method^24,25^. The efficiency of primer pairs for quantitative PCR was determined by making serial dilutions of the cDNA, performing absolute quantitation, plotting C_T_ versus log cDNA dilution, with the slope of the line being used to calculate efficiency^25^. Dissociation curves were obtained to ensure that each primer pair gave a single peak.
